# Oncocytic Change in Thyroid Pathology

**DOI:** 10.3389/fendo.2021.678119

**Published:** 2021-05-03

**Authors:** Sylvia L. Asa, Ozgur Mete

**Affiliations:** ^1^ Department of Pathology, University Hospitals Cleveland Medical Center, Cleveland, OH, United States; ^2^ Department of Pathology, Case Western Reserve University, Cleveland, OH, United States; ^3^ Department of Pathology, University Health Network, Toronto, ON, Canada; ^4^ Department of Laboratory Medicine and Pathobiology, University of Toronto, Toronto, ON, Canada

**Keywords:** thyroid, oncocytes, molecular, neoplasia, Hürthle cell, immunohistochemistry

## Abstract

Oncocytes are cells that have abundant eosinophilic cytoplasm due to the accumulation of mitochondria; they are also known as oxyphils. In the thyroid they have been called Hürthle cells but this is a misnomer, since Hürthle described C cells; for this reason, we propose the use of “oncocyte” as a scientific term rather than an incorrect eponym. Oncocytic change occurs in nontumorous thyroid disorders, in benign and malignant tumors of thyroid follicular cells, in tumors composed of thyroid C cells, and intrathyroidal parathyroid proliferations as well as in metastatic lesions. The morphology of primary oncocytic thyroid tumors is similar to that of their non-oncocytic counterparts but also is complicated by the cytologic features of these cells that include both abundant eosinophilic cytoplasm and large cherry red nucleoli. The molecular alterations in oncocytic thyroid tumors echo those of their non-oncocytic counterparts but in addition feature mitochondrial DNA mutations as well as chromosomal gains and losses. In this review we emphasize the importance of recognition of the spectrum of oncocytic thyroid pathology. The cell of origin, morphologic features including architecture, nuclear atypia and invasive growth, as well as high grade features such as mitoses and necrosis, enable accurate classification of these lesions. The molecular alterations underlying the pathological entity are associated with genetic alterations associated with oncocytic change. The arbitrary cut-off of 75% oncocytic change to classify a lesion as an oncocytic variant brings another complexity to the classification scheme of tumors that frequently have mixed oncocytic and non-oncocytic components. This controversial and often confusing area of thyroid pathology requires thoughtful and cautious investigation to clarify accurate diagnosis, prognosis and prediction for patients with oncocytic thyroid lesions.

## Introduction

The term “oncocyte” was applied to describe cells that have abundant eosinophilic cytoplasm due to the accumulation of mitochondria. The term “oxyphil” has also been used, because of their acidophilic cytoplasm, but this term is less specific since other mechanisms can lead to cytoplasmic acidophilia. “Oncocytosis” derives from the Greek word for swelling or mass, forming the basis for the discipline of oncology. In pathology, oncocytic change is a proliferation of mitochondria within the cell.

This metaplastic change occurs normally with aging in many organs; the prime example is the parathyroid gland where oncocytes are not seen at birth but increase with age (1, 2). The same is true of the pituitary where gonadotrophs, especially those of the pars tuberalis, become more oncocytic with aging (3, 4). Interestingly, there is no documentation of oncocytic change as a feature of normal aging in the thyroid. However, oncocytic change is more frequently seen in response to inflammation where it is clearly a reactive cellular process. It also occurs in tumors, both in the thyroid and in other organs, including endocrine neoplasms (e.g., parathyroid, pituitary, adrenal, pancreas and paraganglia) and non-endocrine neoplasms of salivary and lacrimal glands, kidney and others. In the thyroid, it occurs mainly in follicular epithelial cells (5, 6) but also can occur in C cells and their lesions, and in solid cell nests, the embryological remnants of the ultimobranchial bodies (7).

Oncocytes in the thyroid have been called Hürthle cells but this is a misnomer. Hürthle described the clear cells found associated with thyroid follicles that we now recognize as calcitonin-producing neuroendocrine C cells of the thyroid gland; he did not describe oncocytes (8). In fact, thyroid oncocytes were described by Askanazy (9), who more appropriately deserves the recognition. Sadly, the wrong eponym remains embedded in conventional terminology given its widespread use especially in cytopathology practice. It is most inappropriate, not only because it is incorrect but also because the same phenomenon in other organs is properly described as oncocytic change.

In most sites, oncocytic change has been associated with alterations related to the mitochondrial DNA (10) but also with germline mutations of the *Gene associated with Retinoid-Interferon-induced Mortality (GRIM)-19* (11). Additional molecular alterations have been identified in the various tumors that have oncocytic change.

This review will discuss the phenomenon of oncocytic change in thyroid cells in various pathological conditions and the implications it brings to diagnosis, prognosis and prediction.

## Thyroiditis

**Figure 1:**
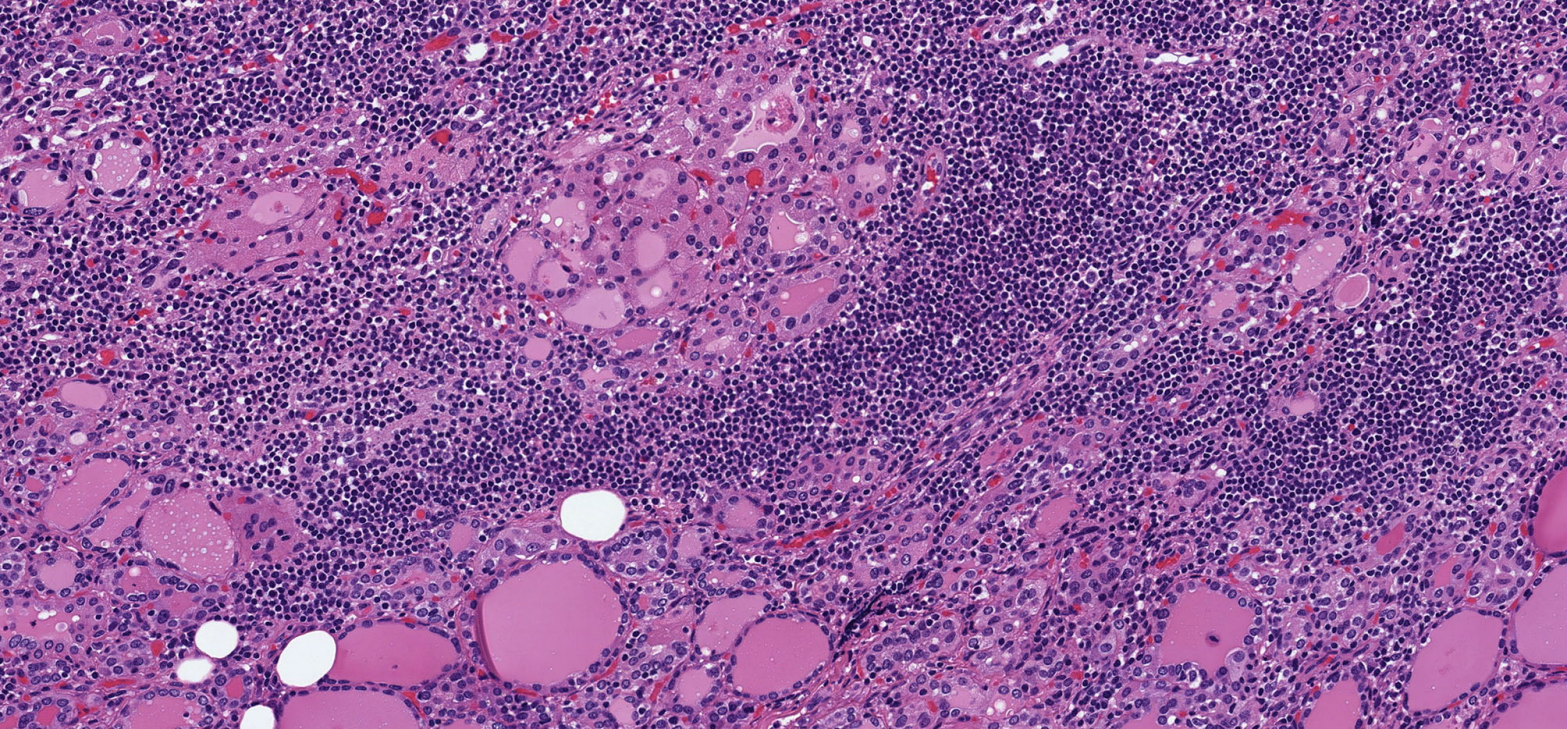
Oncocytic Change in Chronic Lymphocytic Thyroiditis. Chronic lymphocytic thyroiditis is characaterized by a diffuse lymphoplasmacytic infiltrate that forms lymphoid follicles with germinal centers and is associated with oncocytic change of follicular epithelial cells that develop large, granular eosinophilic cytoplasm.

**Figure 2:**
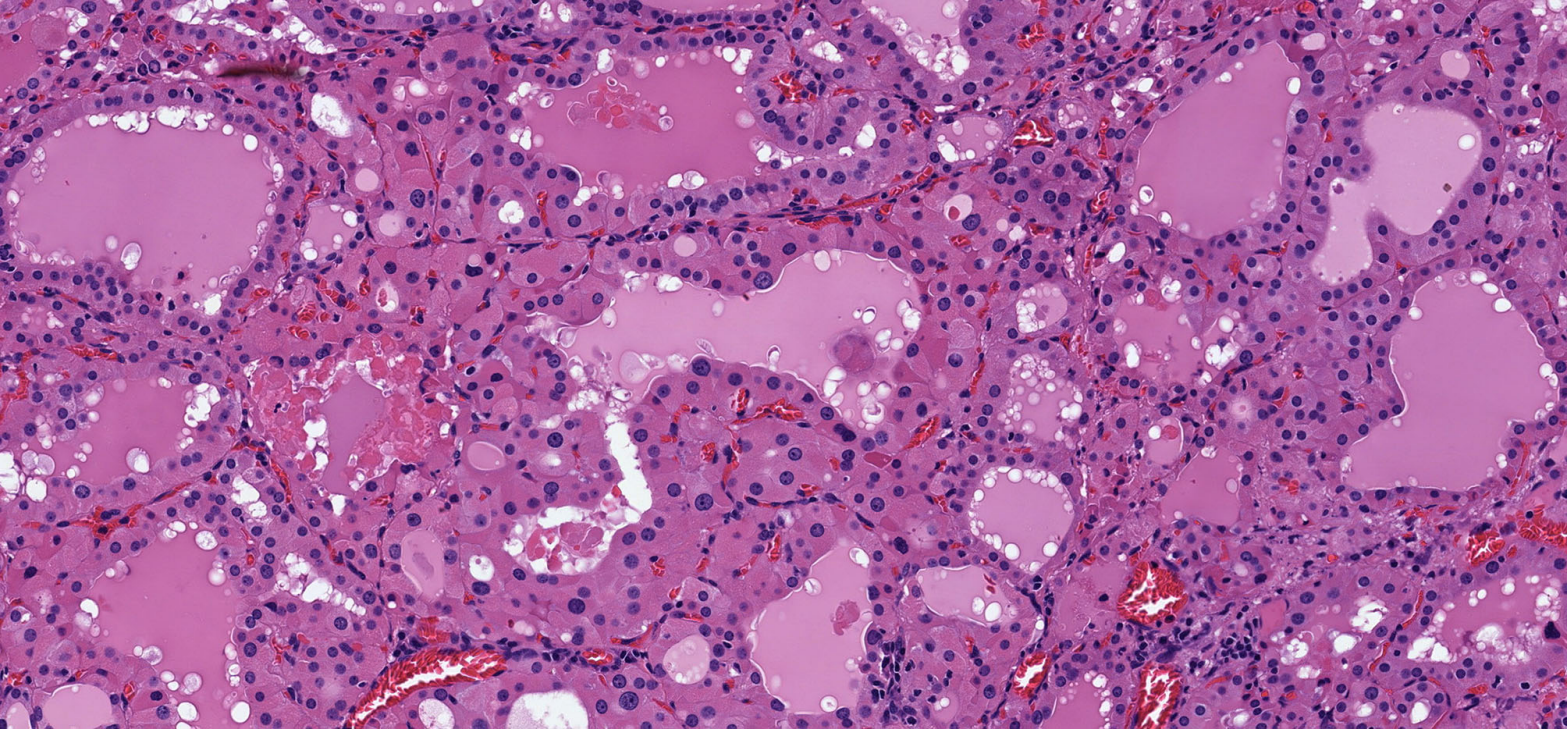
Oncocytic Change in Graves’ Disease. Graves disease is characterized by diffuse papillary hyperplasia with variable degrees of oncocytic change of the hyperstimulated follicular epithelial cells.

Oncocytic change is a characteristic feature of chronic lymphocytic thyroiditis (Hashimoto thyroiditis) (12) (**Figure 1**) and was initially described in the setting of Graves’ disease, another inflammatory condition associated with diffuse hyperplasia of follicular cells (9) (**Figure 2**). The basis for this change is considered to be a response to cellular stress induced by the inflammation or perhaps exhaustion in the hyperstimulated follicular cells of Graves’ disease. These oncocytic follicular cells retain their normal intercellular adhesion and polarity, but they are enlarged and have abundant eosinophilic granular cytoplasm (**Figure 2**). Their nuclei tend to be large but round, and they have prominent cherry-red macronucleoli.

The molecular basis of this change is not as well studied as that in oncocytic neoplasms. In vivo analysis of transgenic mice overexpressing IFNγ in thyroid gland has shown that oncocytes result from increased immunoproteasome expression in a chronic inflammatory milieu (13). Defects in the complex HIII (ubiquinone- cytochrome-c-oxidoreductase) and complex IV (cytochrome-c-oxidase) of the respiratory chain and somatic mitochondrial DNA mutations in NADH dehydrogenase genes and cytochrome c oxidase activity-impairing genes have been found in non-neoplastic oncocytes in parathyroid glands (14, 15), suggesting that these mechanisms are responsible for non-neoplastic, non-clonal oncocytic change.

## Thyroid Follicular Nodular Disease and Adenomas

**Figure 3:**
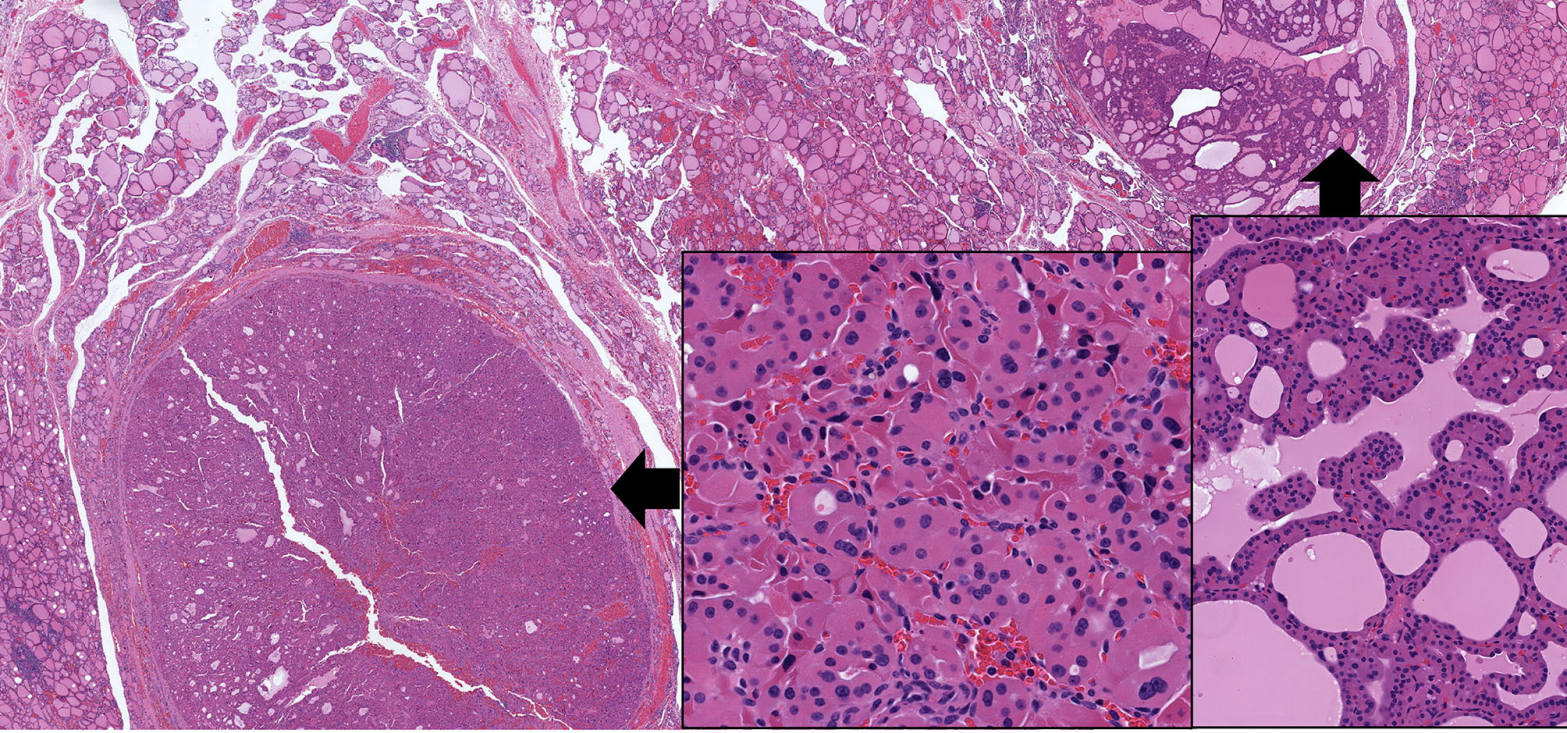
Oncocytic Change in Follicular Nodular Disease of Thyroid. Multifocal benign proliferations of thyroid follicular epithelium have variable architecture and cytology. In some instances, they can exhibit focal or diffuse oncocytic change; in this example, the gland has two adjacent lesions, one with follicular architecture (left) and one with papillary architecture (right), both showing extensive oncocytic cytology (insets) but retaining the uniform, round and homogenous nuclear morphology of benign thyroid follicular epithelium.

**Figure 4:**
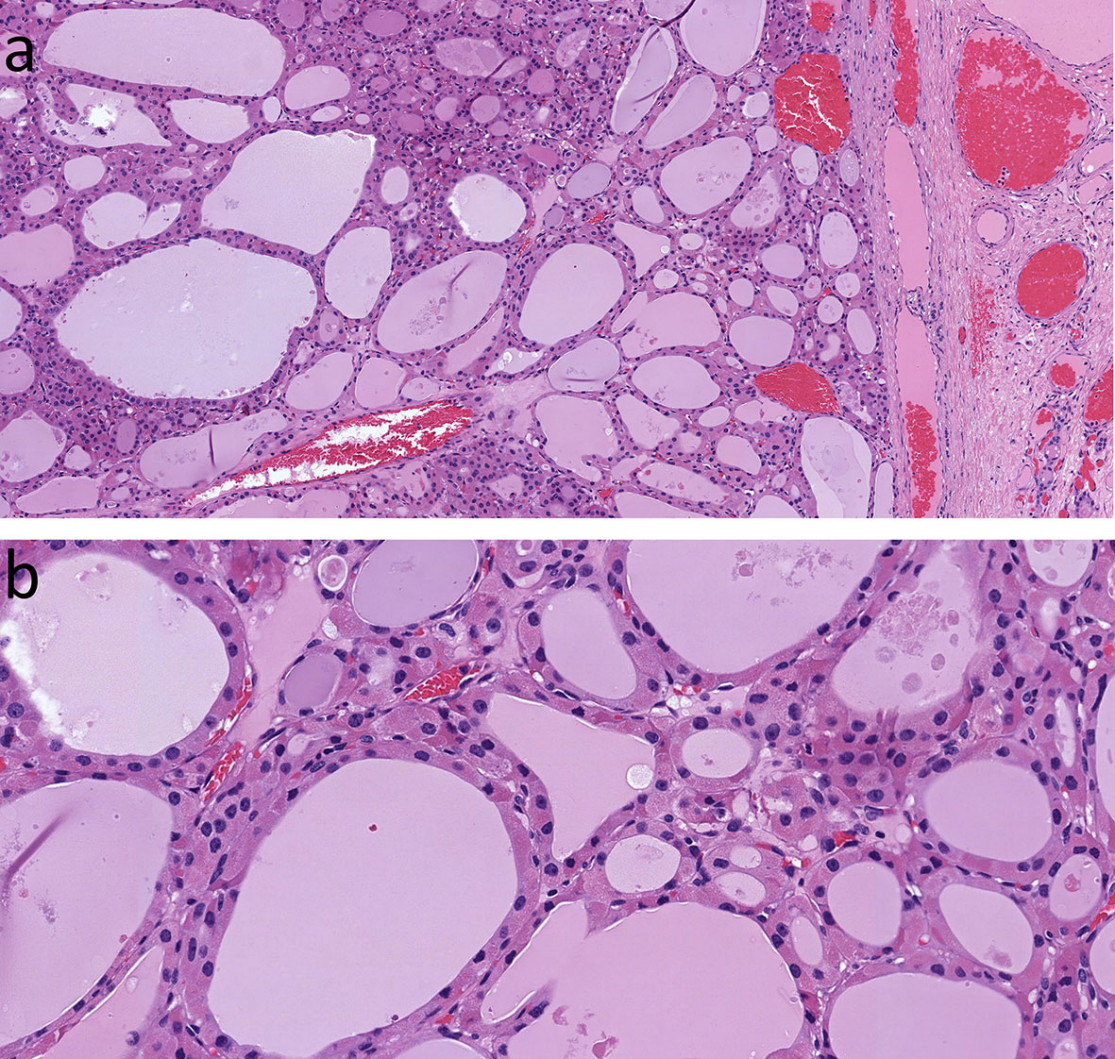
Oncocytic Follicular Thyroid Adenoma. This large, solitary neoplasm of thyroid follicular cells with follicular architecture is well delineated **(A)**, and has bland nuclear morphology **(B)** with no evidence of invasive behavior at the capsule.

Oncocytic change is seen in follicular nodular disease (**Figure 3**) and in thyroid adenomas (**Figure 4**). It can be diffuse or focal (16, 17). The presence of oncocytic change results in nuclear features that are not “normal”; however, the presence only of enlarged nuclei with prominent cherry red macronucleoli should not be of concern in these tumors. Other nuclear atypia, such as irregular nuclear contours, peripheral margination of chromatin, nuclear pseudoinclusions, and crowding with overlap and moulding should indicate malignancy as in other follicular thyroid neoplasms (12, 18). Of course, the presence of invasive behaviour, into or beyond tumor capsule, lymphatics and/or blood vessels, is also universally accepted as a criterion for classification of an oncocytic thyroid neoplasm as malignant.

These tumors have mitochondrial DNA alterations but also have chromosomal gains and losses that are not seen in non-oncocytic tumors, and that do not often differ between benign and malignant tumors (19); interestingly adenomas cluster with minimally invasive carcinomas and only the widely invasive malignancies have a distinct profile including activation of the PIK3CA-Akt-mTOR and Wnt/β-catenin pathways (19).

The diagnosis of oncocytic follicular adenoma has been questioned, since some authors have found that oncocytic follicular lesions called benign subsequently behave in a malignant fashion (20). This may be attributed to failure to recognize the nuclear atypia of malignancy in these neoplasms (12, 18, 21, 22), but also partly to common challenges in the identification of invasive growth in follicular neoplasms (23–26).

## Follicular-Patterned Differentiated Thyroid Carcinomas

Follicular-patterned differentiated thyroid carcinomas include follicular carcinomas and follicular variant papillary thyroid carcinomas (FVPTC). They are distinguished based on the degree of nuclear atypia, which is considered to be mild in follicular carcinoma, and is more evident in FVPTC (27, 28). Tumors that used to be classified as FVPTC has been recognized to include two distinct tumors, the invasive variant that frequently harbors *BRAFV600E* mutations and is more akin to classical papillary thyroid carcinoma, and the encapsulated type, which is an expansile lesion that resembles follicular carcinoma grossly and at low magnification.

Molecular analyses have shown that follicular carcinoma and encapsulated follicular variant papillary carcinoma are both RAS-like lesions (29, 30) that are now classified as (i) minimally invasive with tumor capsular invasion only, (ii) angioinvasive but with no widely invasive growth, and (iii) widely invasive and often angioinvasive, with more aggressive behavior (31). Indeed, the distinction between follicular carcinoma and the encapsulated FVPTC is in question since the management approach to both entities overlaps (32, 33). Noninvasive tumors without nuclear atypia are classified as adenomas whereas those with nuclear atypia are Non-Invasive Follicular Tumors with Papillary-like nuclei (NIFTP) (34).

**Figure 5:**
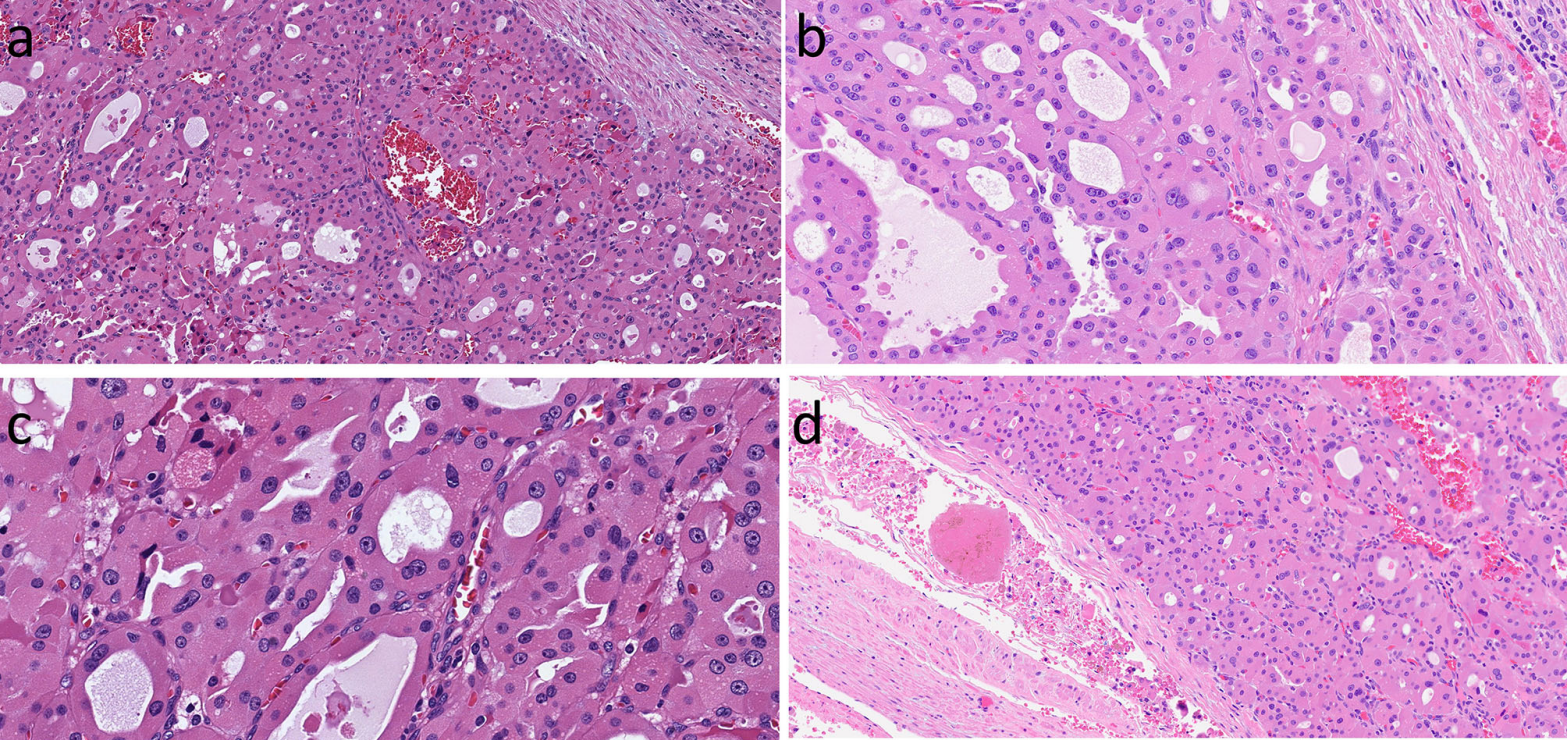
Oncocytic Follicular-Patterned Differentiated Thyroid Carcinoma. Follicular neoplasms composed of oncocytic follicular cells can exhibit local invasion into the tumor capsule **(A)**. They may have nuclear atypia including irregular contours, moulding, clearing of nucleoplasm and grooves **(B, C)** in addition to the prominent nuleoli of oncocytic cells. There may be lymphatic invasion that is associated with regional lymph node spread (not shown) and/or vascular invasion with associated intravascular thrombus **(D)** that predicts systemic metastases.

Follicular patterned neoplasms including follicular carcinoma and FVPTC, can exhibit oncocytic cytology (**Figure 5**). When classified using rigid criteria and in the setting of exclusive follicular growth and absence of dedifferentiation, the oncocytic counterparts of follicular carcinoma and encapsulated FVPTC represent thyroid neoplasms classified as one type of “oncocytic (Hürthle) cell carcinoma” (31). As in non-oncocytic tumors, these neoplasms are classified as (i) minimally invasive oncocytic carcinoma (tumor capsular invasion only), (ii) angioinvasive encapsulated oncocytic carcinoma (angioinvasion with no widely invasive growth), and (iii) widely invasive oncocytic carcinoma (often angioinvasive) (31). Oncocytic lesions that have nuclear atypia but lack invasion fall into the category of Non-Invasive Follicular Tumor with Papillary-like nuclei (NIFTP) (35); however, this remains controversial since it has been reported that oncocytic tumors with follicular architecture and nuclear atypia that lack overt invasion in sections examined can metastasize (12, 18, 20–22).

Several molecular analyses of oncocytic tumors have classified these lesions only based on their oncocytic cytology and failed to consider other relevant criteria of more aggressive thyroid carcinomas such as predominant solid growth, necrosis and high mitotic activity (>3 per 2mm^2^), creating potential challenges that may explain relatively lower rates of RAS-like molecular profiles and higher rates of high-risk molecular alterations in these studies (19, 36, 37). These findings emphasize the importance of the accurate distinction of a poorly differentiated oncocytic thyroid carcinoma from a well differentiated oncocytic carcinoma based on growth pattern and proliferation. Nevertheless, a consistent finding is that oncocytic follicular thyroid carcinomas have mitochondrial DNA mutations (10, 38, 39) as well as chromosomal gains and losses, and occasional *EIF1AX, RAS, PTEN* and *PIK3CA* mutations (19, 37, 40).

The requirement to distinguish oncocytic from non-oncocytic follicular carcinomas stems partly from the spectrum of molecular alterations but also from the relative lack of uptake of radioactive iodine (36, 41). For instance, only 38% of patients with metastatic disease had iodine uptake in one study (42). However, it is critical to also assess and account for morphological dedifferentiation to poorly differentiated thyroid carcinomas; as with molecular studies, a wide range of diagnostic heterogeneity may contribute to the reduced response to radioactive iodine therapy in reports of clinical outcomes of “Hürthle cell carcinoma”.

## Papillary Thyroid Carcinoma

Since oncocytic FVPTCs are discussed above, this section is primarily focused on oncocytic papillary thyroid carcinomas with classic and solid growth.

**Figure 6:**
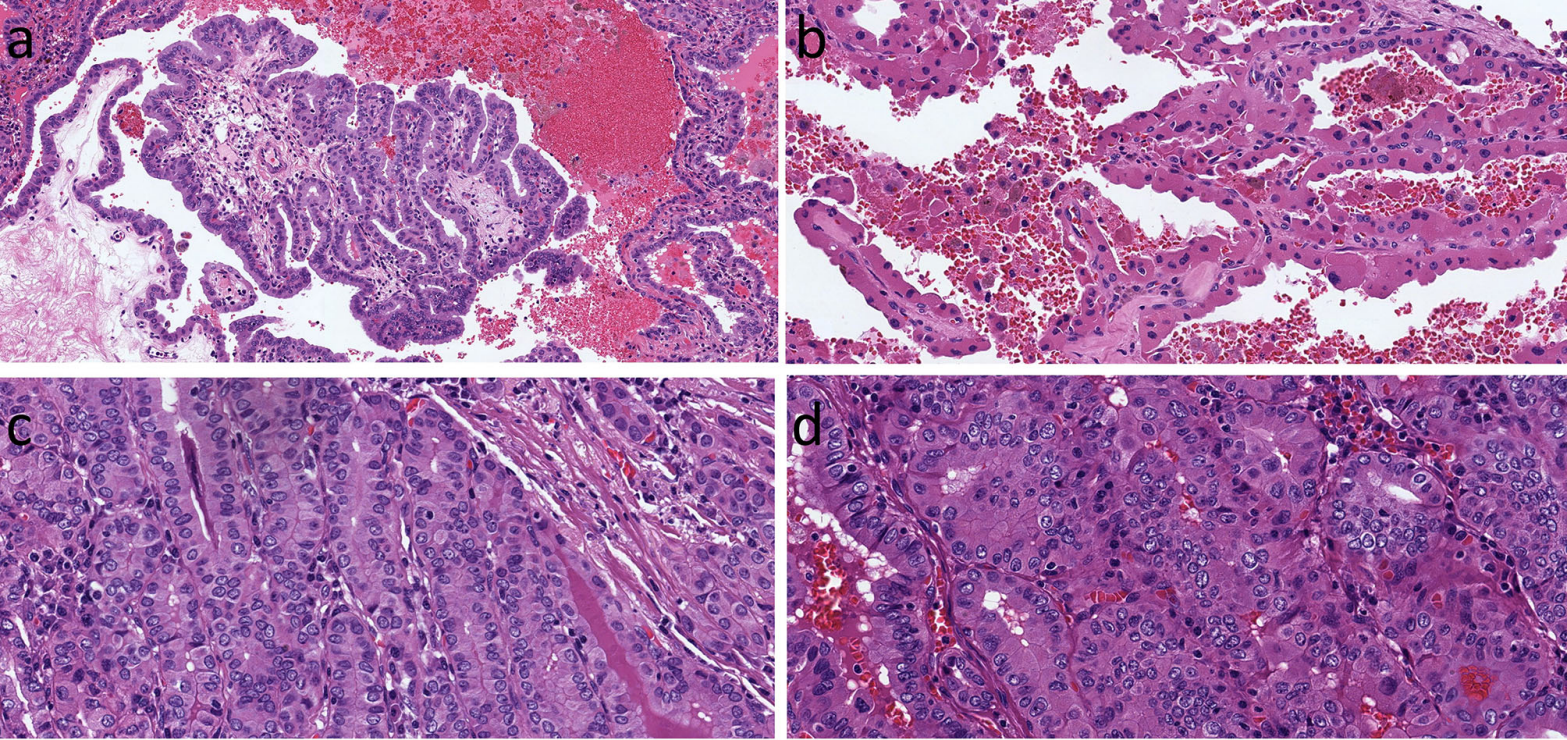
Oncocytic Classic Papillary Thyroid Carcinoma. A classical papillary thyroid carcinoma has oncocytic cytology of the columnar cells lining a complex papilla **(A)**. In this example, the oncocytic cells are more polygonal and cuboidal **(B)**. When the oncocytic cells are crowded, there is a superficial resemblance to tall cell papillary thyroid carcinoma, but careful inspection shows that the height-to-width ratio does not exceed 3:1 **(C)**. The oncocytic cells can have variable shapes and sizes **(D)**, however in all of these examples, the nuclear features of papillary caricnoma are readily appreciated.

**Figure 7:**
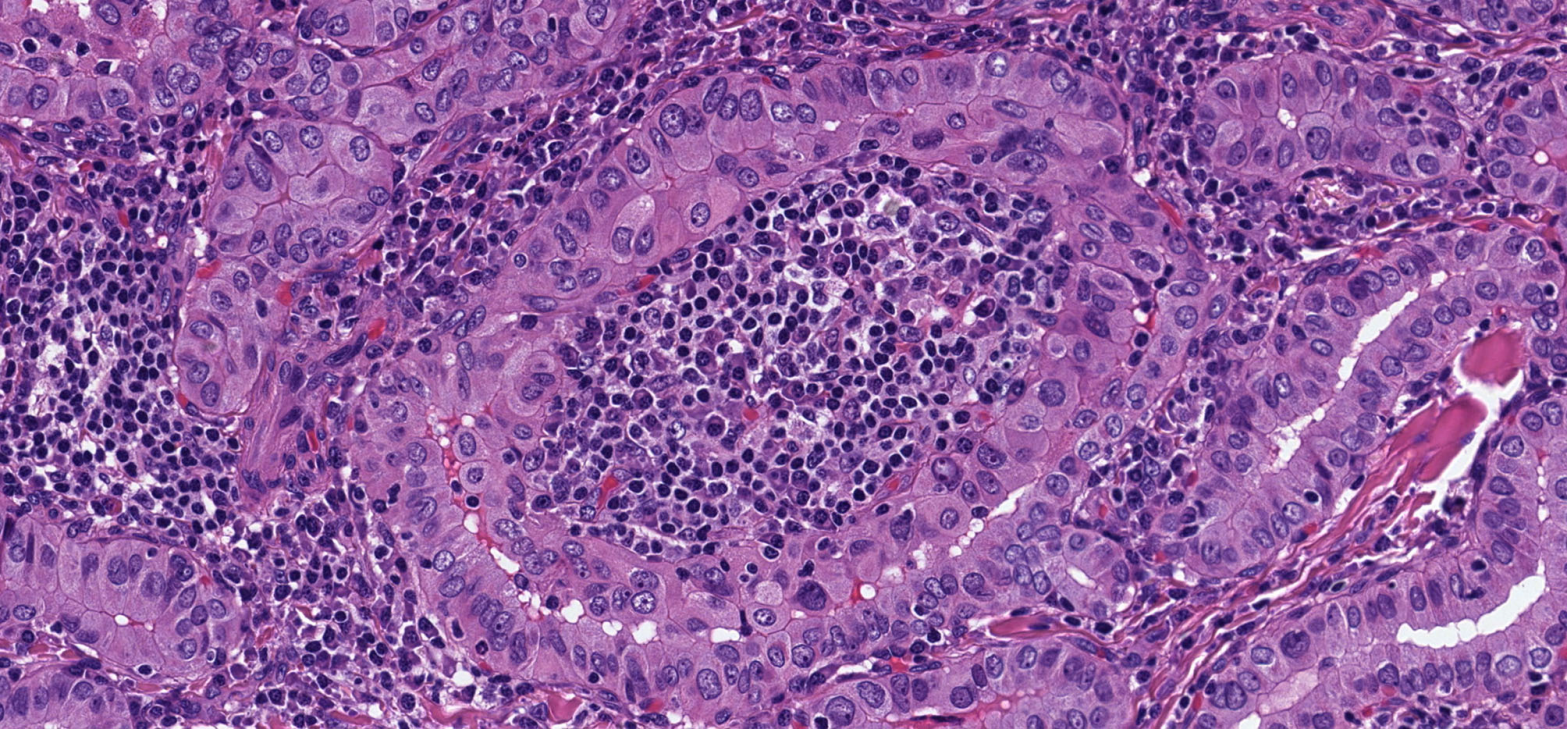
Warthin-Like Papillary Thyroid Carcinoma. This oncocytic papillary thyroid carcinoma has a striking lymphoplasmacytic infiltrate in the stroma that yeilds a resemblance to Warthin tumor of salivary glands.

Papillary thyroid carcinoma with complex papillary architecture, known as the classical variant, and infiltrative carcinomas with predominant follicular architecture, known as the infiltrative follicular variant, represent the BRAF-like family of thyroid carcinomas that have an infiltrative rather than expansile pattern of growth and florid nuclear atypia. The presence of oncocytic change in these thyroid carcinomas is not appreciated as much as in follicular lesions of the thyroid. However, there are examples of papillary thyroid carcinomas with papillary architecture including the presence of complex papillae with fibrovascular cores and florid nuclear features of papillary carcinoma in which the tumor cells have oncocytic cytoplasm and the nuclei may also contain cherry red macronucleoli (**Figure 6**) (18, 43). This morphologic variant of papillary thyroid carcinoma has sometimes been confused with tall cell papillary carcinoma which usually lacks the characteristic cherry red macronucleoli; this underscores the importance of verifying a height-to-width ratio that exceeds 3:1 (44). A unique variant of oncocytic classical variant papillary thyroid carcinoma is known as the “Warthin-like” variant because it consists of tumor cells with oncocytic change but more importantly has a prominent lymphoid stroma that makes it resemble the Warthin tumor of salivary gland (45, 46) (**Figure 7**).

To date, oncocytic classical variant papillary thyroid carcinomas have not been shown to behave differently than other classical variant papillary carcinomas (47). They tend to be infiltrative; they metastasize to lymph nodes and they harbor *BRAFV600E* mutations or *RET/PTC* gene rearrangements (21, 22, 48).

Oncocytic papillary thyroid carcinomas also feature occasional tumors with extensive solid growth (often >30% of the tumor volume); solid growth also encompasses trabecular and insular/nested architecture. These oncocytic forms of solid variant papillary thyroid carcinomas can be difficult to recognize as papillary carcinomas due to the difficulty of appreciating the extent of nuclear atypia of papillary thyroid carcinoma in oncocytes; however, there is often a papillary and/or follicular growth component as well. The differential diagnosis is oncocytic poorly differentiated carcinoma, as the solid growth may indicate dedifferentiation, but the distinction is based on lack of necrosis and mitoses that are characteristic of high-grade malignancies. Importantly, the differential diagnosis of oncocytic solid variant papillary thyroid carcinoma includes oncocytic medullary thyroid carcinoma (49, 50) and other oncocytic neuroendocrine neoplasms including but not limited to oncocytic parathyroid neoplasms and thyroid paragangliomas with oncocytic change. It should be noted that solid growth in papillary thyroid carcinomas occurs in both RAS-like and BRAF-like tumors, as well as carcinomas with oncogene fusions that fall into these molecular subgroups.

## Poorly Differentiated Carcinoma

**Figure 8:**
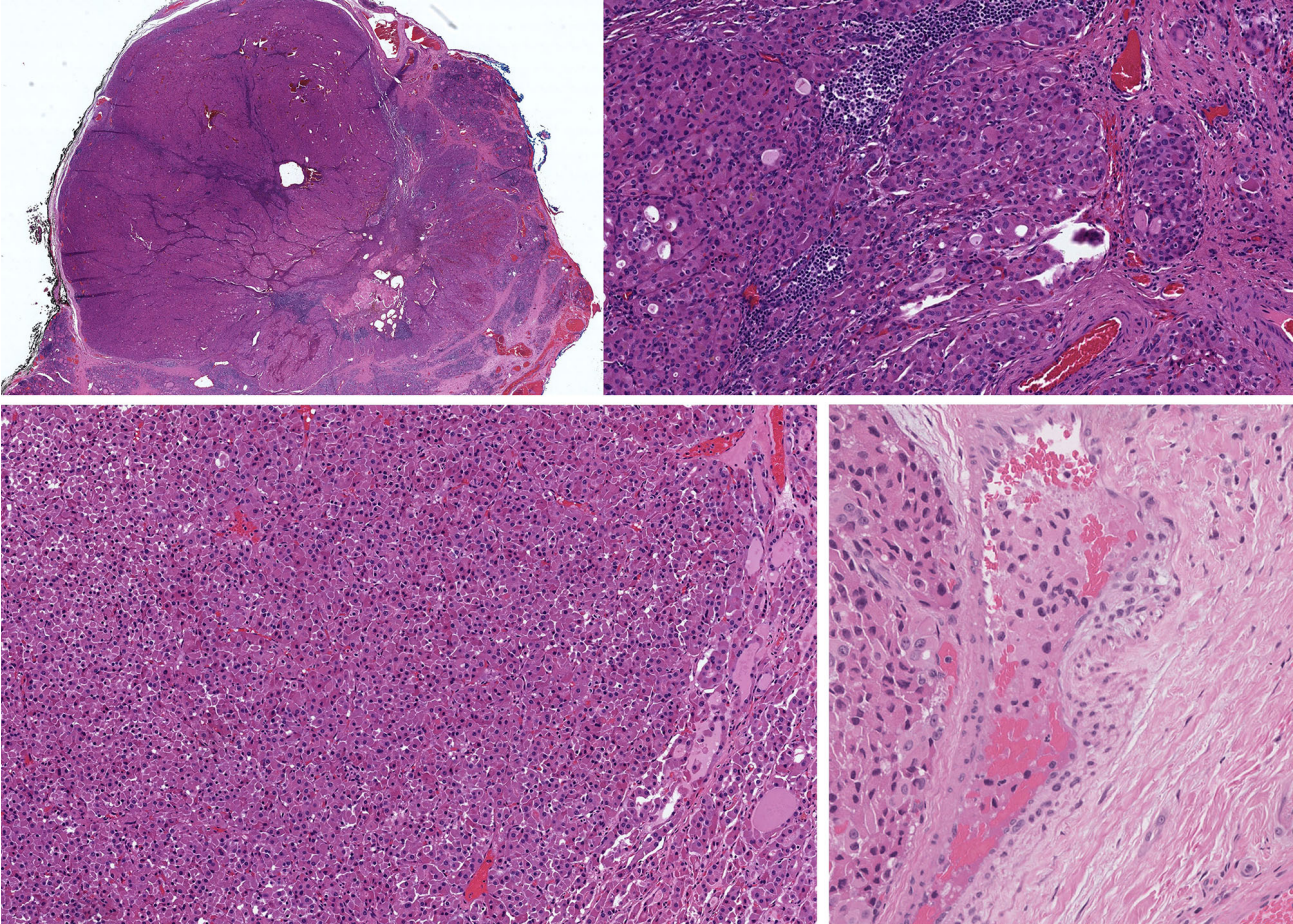
Oncocytic Poorly Differentiated Thyroid Carcinoma. These oncocytic tumors are widely invasive (top left) with multifocal capsular invasion (top right) and prominent solid architecture with only focal residual follicles (bottom left). Vascular invasion is readily identified (bottom right).

**Figure 9:**
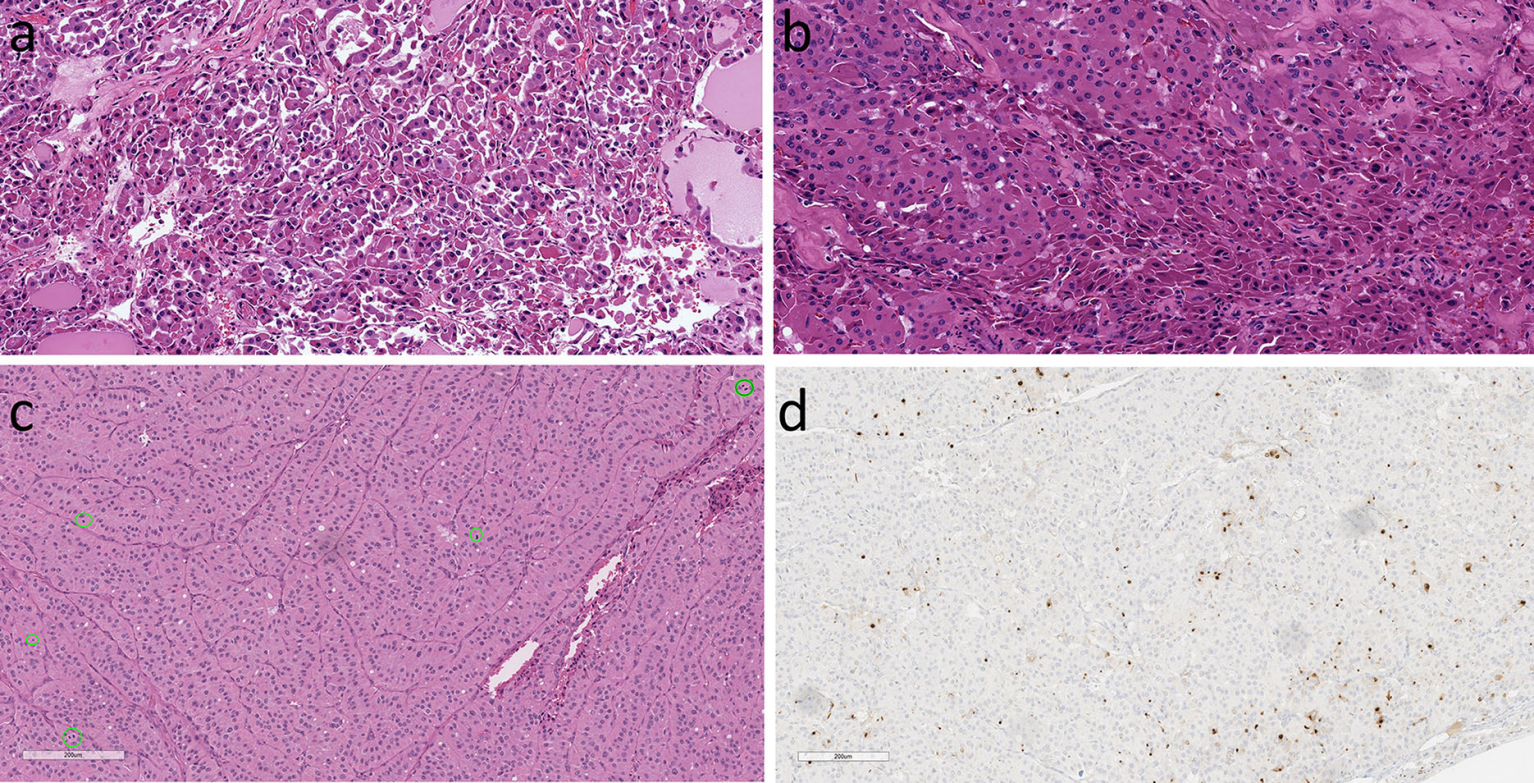
Oncocytic Poorly Differentiated Thyroid Carcinoma. These aggressive tumors are characterized by discohesive solid growth **(A)** and focal tumor cell necrosis **(B)** with increased mitotic activity. that exceeds 3 per 2mm^2^ **(C)** and loss of thyroglobulin expression **(D)**.

A subset of classical “Hürthle cell carcinoma” actually represents a poorly differentiated tumor with predominant solid growth and widely invasive behaviour (**Figures 8**, **9**), often with a well delineated nidus (16, 17, 38). These tumors tend to exhibit focal necrosis and have prominent mitoses. These are the features of poorly differentiated thyroid carcinoma, also known as “insular thyroid carcinoma” because of the solid nesting growth pattern (51). Immunohistochemical biomarkers supportive of dedifferentiation can help to distinguish poorly differentiated thyroid carcinoma, including focal areas of dedifferentiation indicative of tumor progression (52). The oncocytic variants of this entity tend to be very aggressive and, like their non-oncocytic counterparts, rarely respond to therapy with radioactive iodine. In addition to the mitochondrial DNA mutations, chromosomal gains and losses, and occasional mutations characteristic of differentiated thyroid carcinomas, they harbor *TERT* promoter mutations and occasional mutations of *ATRX* and/or *DAXX* (19, 37). As in other thyroid tumors, *TERT* promoter mutation portents a more aggressive clinical course with distant metastatic spread, and reduced iodine uptake. The clinical relevance of recognizing this tumor classification has been highlighted by a recent study showing a distinctive immune-related gene expression profile of oncocytic poorly differentiated thyroid carcinomas, not only confirming that this is a more aggressive cancer subtype but also pointing to a potential role for immunotherapy in the management of these high-grade malignancies (53).

## Medullary Thyroid Carcinoma

**Figure 10:**
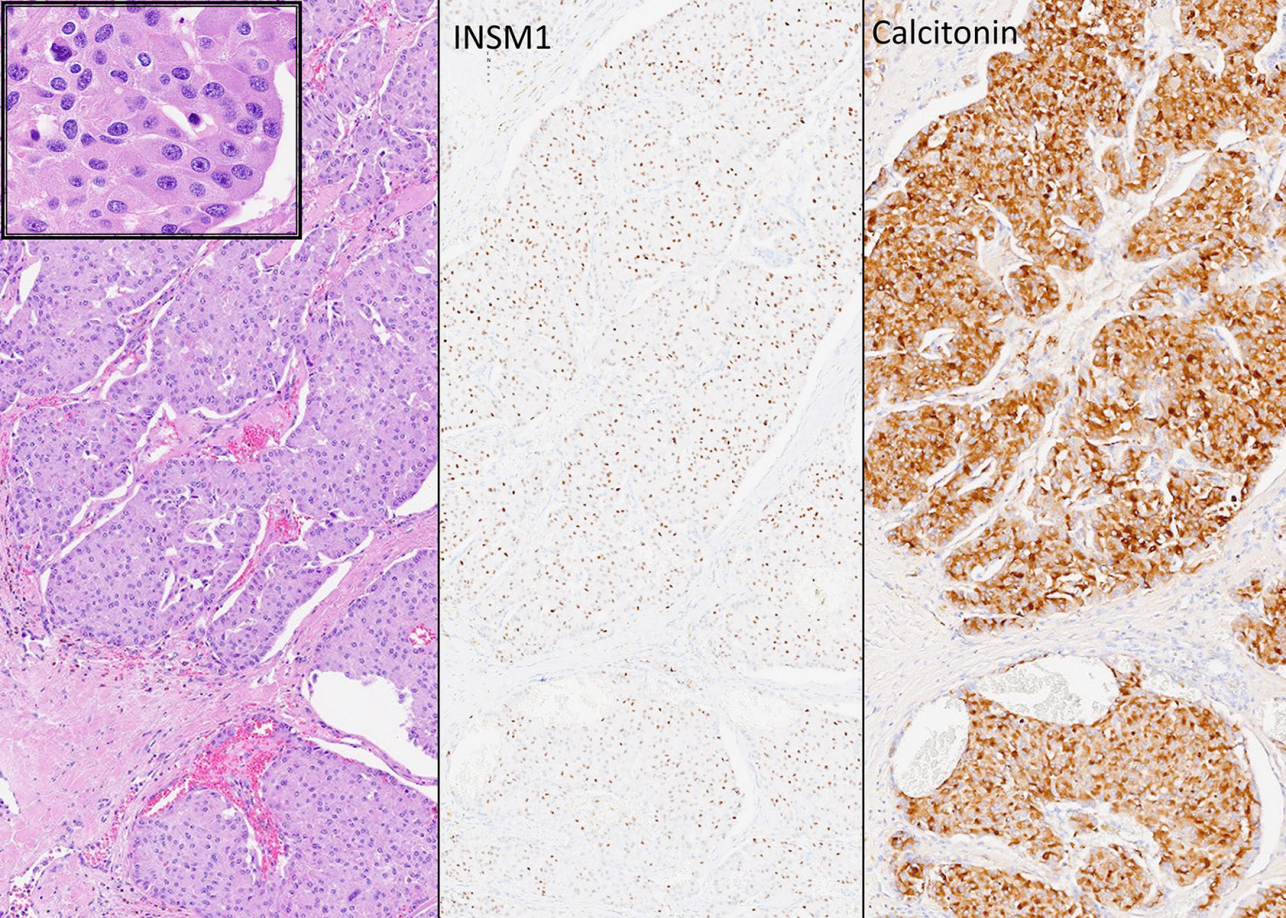
Oncocytic Medullary Thyroid Carcinoma. Medullary thyroid carcinoma can have oncocytic cyology (left and insert). These rare tumors are easily misdiagnosed as oncocytic follicular tumors but immunohistochemistry confirms the expression of INSM1 (middle) and calcitonin (right) as well as chromogranin and monoclonal CEA (not shown) that distinguishes these tumors from other calcitonin-expressing neuroendocrine neoplasms.

The existence of oncocytic medullary thyroid carcinoma is well documented (1, 12, 18) (**Figure 10**) but this lesion is often misdiagnosed. It occurs only rarely and requires a high degree of clinical suspicion, since oncocytic tumors in thyroid are often rapidly diagnosed as “Hürthle cell carcinoma”. Ironically, in this situation, that terminology would be technically the correct eponym, but the implications of a diagnosis of medullary thyroid carcinoma, including potential germline predisposition, identification of calcitonin and CEA rather than thyroglobulin for surveillance, and lack of a role for radioactive iodine therapy all would be missed. Lack of cellular cohesiveness and the presence of basophilic to amphophilic cytoplasmic granularity are features that should suggest the possibility of an oncocytic medullary thyroid carcinoma. As with the non-oncocytic counterpart, confirmation of this diagnosis should be based on the identification of diffuse positivity for monoclonal CEA as well as reactivity for calcitonin and/or calcitonin gene-related peptide (CGRP). Importantly, some medullary thyroid carcinomas lack calcitonin expression but can express CGRP whereas others, usually more aggressive tumors that show dedifferentiation can be negative for both calcitonin and CGRP while they preserve diffuse staining with monoclonal antibodies to CEA. A diagnostic pitfall can occur due to the “stickiness” of oncocytes that can show nonspecific background staining (12) resulting in artefactual reactivity for calcitonin. The importance of CEA as a biomarker is also emphasized by the fact that calcitonin and CGRP can be expressed in various other neuroendocrine neoplasms, including those originating in lung, pancreas, head and neck, as well as rare examples of paragangliomas and parathyroid carcinomas (54).

Little is known about the molecular alterations that underlie the oncocytic variant in addition to the frequent *RET* mutations and less common *RAS* mutations that occur in non-oncocytic medullary thyroid carcinomas.

## Metastatic and Invasive Oncocytic Tumors of Non-Thyroid Origin

The presence of oncocytic change is not unique to thyroid, as indicated above. Therefore, the identification of an oncocytic tumor in thyroid must also include consideration of a possible metastatic oncocytic neoplasm, including renal and other primary sites, as well as an infiltrating oncocytic tumor, such as a parathyroid, salivary gland or thymic tumor arising in the region of the thyroid.

## Conclusions

This review has identified the unique features of oncocytic cells and tumors in the thyroid but has also emphasized the importance of recognizing the similarities of oncocytic pathologies in this gland with those of their non-oncocytic counterparts.

A critical issue to remember is that most oncocytic lesions can be diffusely or focally oncocytic with variable non-oncocytic components (16, 17). Interestingly, thyroid tumors are classified as “oncocytic” when 75% of neoplastic cells show this alteration whereas in other body locations, such as kidney and salivary gland, stricter criteria are applied and oncocytic tumors are more homogenous (16). Thyroid tumors with less than 75% oncocytic change are classified as tumors of their given type with focal oncocytic change, but this cut-off is arbitrary and there is no evidence to support this definition.

The data point to heterogeneity of oncocytic thyroid lesions and significant overlap between oncocytic and non-oncocytic lesions, both morphologically and at the molecular level, with additional complexities of the morphologic and molecular alterations of oncocytic change. This complexity has created confusion both for pathologists who must classify these lesions and for clinicians who must treat the patients. Thus, we emphasize the importance of recognition of the spectrum of oncocytic change in thyroid pathology, based on cell of origin, morphology that includes architecture, nuclear atypia and invasion, as well as high grade features such as mitoses and necrosis, and the molecular alterations underlying both the pathological entity as well as the oncocytic change. Only with such a thoughtful and cautious approach will it be possible to clarify accurate diagnosis, prognosis and prediction for patients with oncocytic thyroid pathology.

## Author Contributions 

SA and OM: substantial contributions to conception and design, acquisition of data or analysis and interpretation of data, drafting the article or revising it critically for important intellectual content. All authors contributed to the article and approved the submitted version.

## Conflict of Interest

The authors declare that the research was conducted in the absence of any commercial or financial relationships that could be construed as a potential conflict of interest.
